# Monitoring ultrafast vibrational dynamics of isotopic molecules with frequency modulation of high-order harmonics

**DOI:** 10.1038/s41467-018-03568-3

**Published:** 2018-03-16

**Authors:** Lixin He, Qingbin Zhang, Pengfei Lan, Wei Cao, Xiaosong Zhu, Chunyang Zhai, Feng Wang, Wenjing Shi, Muzi Li, Xue-Bin Bian, Peixiang Lu, André D. Bandrauk

**Affiliations:** 1 Wuhan National Laboratory for Optoelectronics and School of Physics, Huazhong University of Science and Technology, 430074 Wuhan, China; 2 State Key Laboratory of Magnetic Resonance and Atomic and Molecular Physics, Wuhan Institute of Physics and Mathematics, Chinese Academy of Sciences, 430071 Wuhan, China; 3 University of Chinese Academy of Sciences, 100049 Beijing, China; 4 Laboratory of Optical Information Technology, Wuhan Institute of Technology, 430205 Wuhan, China; 5 Laboratoire de chimie théorique, Département de Chimie, Université de Sherbrooke, Sherbrooke, J1K 2R1 Quebéc Canada

## Abstract

Molecules constituted by different isotopes are different in vibrational modes, making it possible to elucidate the mechanism of a chemical reaction via the kinetic isotope effect. However, the real-time observation of the vibrational motion of isotopic nuclei in molecules is still challenging due to its ultrashort time scale. Here we demonstrate a method to monitor the nuclear vibration of isotopic molecules with the frequency modulation of high-order harmonic generation (HHG) during the laser-molecule interaction. In the proof-of-principle experiment, we report a red shift in HHG from H_2_ and D_2_. The red shift is ascribed to dominant HHG from the stretched isotopic molecules at the trailing edge of the laser pulse. By utilizing the observed frequency shift, the laser-driven nuclear vibrations of H_2_ and D_2_ are retrieved. These findings pave an accessible route toward monitoring the ultrafast nuclear dynamics and even tracing a chemical reaction in real time.

## Abstract (web summary)

Previous studies on high harmonic generation from molecules have been used to identify the spectral properties and orbital contributions. Here the authors measure the isotopic effects in the energy shift of the HHG spectra caused by the nuclear motion of the molecules.

## Introduction

Since Soddy first suggested the existence of isotopes in 1913^1^, isotopes have drawn a great deal of attention due to its application in the fields of physics, chemistry, biomedicine, and geology. Generally, isotopes with different nuclear masses could change the energy levels within isotopic atoms and molecules, thus lead to a frequency shift in the atomic or molecular spectrum^2^, which has been widely used to identify the species of the isotopes and to investigate the static structure of the isotopologues. Moreover, for isotopic molecules, the vibrational modes depend sensitively on the masses of its constituent isotopic atoms, which provides an important method to determine the mechanism of a chemical reaction via the kinetic isotope effect^3,4^, namely, the fact that heavier isotopes tend to react more slowly than lighter ones. However, a real-time measurement of the motions of the isotopic atoms in molecule (molecular vibration) is a long-standing challenge over the last century, due to the awesome rapidity of the molecular vibration.

**Fig. 1:**
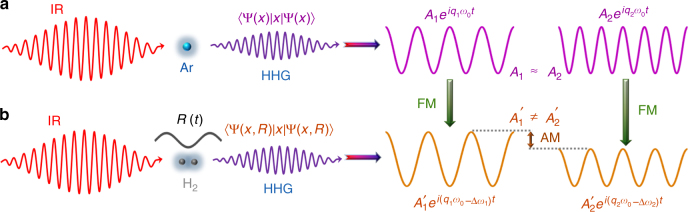
Sketch of AM and FM in MHOHG. **a** Schematic diagram of HHG from atoms. The harmonic spectrum generated with atom is composed by series regular odd-order harmonics. For different harmonics (*q*_1_ and *q*_2_) in the plateau region, the harmonic intensities are comparable (*A*_1_ ≈ *A*_2_). **b** Schematic diagram of HHG from molecules. For molecules, the laser-driven nuclear motion introduces an additional degree of freedom and will modulate the MHOHG. As demonstrated in refs. ^27–29^, the harmonic intensity in the plateau is approximately proportional to the square of modulus of the nuclear correlation function, which depends sensitively on the traveling time of the electron in the continuum. For different harmonics *q*_1_ and *q*_2_, the traveling times of the electrons are different, thus leading to an AM in the spectrum (*A*′_1_ ≠ *A*′_2_). On the other hand, the laser-driven nuclear motion will enhance the ionization rate and thus strengthen the harmonic emission at the trailing edge of the laser pulse. Due to the laser-driven nonadiabatic effect, harmonics dominated at the trailing edge will emerge a FM (red shift) in the spectrum (*q*_1_*ω*_0_ → *q*_1_*ω*_0_ − Δ*ω*_1_, *q*_2_*ω*_0_ → *q*_2_*ω*_0_ − Δ*ω*_2_)

Recent advances in strong-field physics have provided efficient approaches to probe both the molecular structure and dynamics using the table-top laser. These new methods rely on the recollision of an electron, removed from the molecule by a strong laser field, with its parent ion. The molecular structure and dynamics are encoded in the amplitude and phase of the emitted high-order harmonics. It stimulates the development of high-order harmonic spectroscopy (HHS)^5–9^ as an emerging tool for ultrafast detection with femtosecond to attosecond time resolutions. Apart from HHS, some other techniques based on strong-field ionization, such as photoelectron holography and photoelectron diffraction^10–17^, and so on, have also been demonstrated to image the molecular structure and dynamics. Up to now, many works have been carried out to investigate the effects of nuclear motion in strong-field ionization^18–21^ and molecular high-order harmonic generation (MHOHG)^22–26^. In 2005, Lein showed theoretically that the laser-driven nuclear motion will introduce an amplitude modulation (AM) (see Fig. 1) in harmonic signals via the nuclear autocorrelation function^27^, which denotes the overlap between the initial and time-dependent nuclear wave function that evolves from the moment of ionization until the recollision. By analyzing the AMs in high-order harmonic generation (HHG) from isotopic molecules (H_2_ and D_2_), the intracycle nuclear dynamics has been theoretically predicted^27^ and experimentally detected^28,29^. Nevertheless, this method is restricted because the propagation and other inherent physical factors, such as two-center interference^27,30^ and energy-dependent rescattering cross sections^31^, may affect the harmonic intensity. Moreover, in the presence of intense lasers, the nuclear motion will lead to larger internuclear distances *R* and a decrease in the ionization potential *I*_p_^18–20^, which can result in an increase in the ionization rate and thus a strong AM in MHOHG. These factors complicate the retrieval of nuclear dynamics by AM. Apart from AM, frequency modulation (FM) is an alternative way commonly used in various applications, e.g., signal processing and telecommunications. By considering the frequency shift in the atomic spectrum of isotopes, it stimulates us to ask whether the nuclear motion in intense laser fields can induce a frequency shift in the MHOHG spectrum (see Fig. 1). Compared to AM, FM is more stable and insensitive to the laser parameters provided that the ionization saturation is avoided and the pulse length is properly adopted. It thus can provide an alternative powerful way to identify the nuclear dynamics. After the prediction by Bian and Bandrauk in ref. ^32^, FM has received a lot of attention in theoretical studies^31,33–35^. However, the FM in isotopic MHOHG has never been observed in experiment and the measurement of nuclear motion based on the FM is not addressed.

In the present work, we report the experimental observation of FM in MHOHG from isotopic molecules H_2_ and D_2_. High-order harmonics generated from isotopic molecules show obvious red shift with respect to those from Ar atom. The red shift is demonstrated to originate from the laser-induced nuclear motion of isotopic molecules, which strengthens harmonic emission at the trailing edge of the laser pulse. From the observed frequency shift, the nuclear motions of H_2_ and D_2_ are successfully retrieved, which agree well with the calculations from non-Born–Oppenheimer time-dependent Schrödinger equation (NBO–TDSE).

## Results

### Experimental observation of FM

**Fig. 2:**
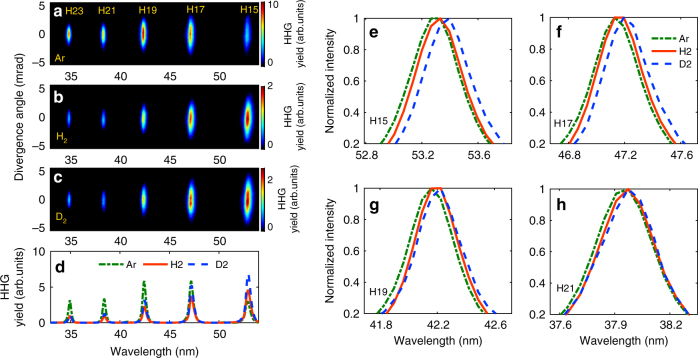
Experimentally measured harmonic signals. **a**–**c** are the spatially resolved harmonic spectra of Ar, H_2_, and D_2_. **d** shows the spatially integrated HHG signals for the spectra in **a** (dash-dotted line), **b** (solid line), and **c** (dashed line), respectively. For clarity, the dash-dotted line is multiplied by a factor of 0.2. **e**–**h** are the normalized harmonic signals of H15-H21 for Ar (dash-dotted line), H_2_ (solid line), and D_2_ (dashed line), respectively. Here, the laser intensity is 1.5 × 10^14^ W cm^−2^ and the pulse duration is 30 fs

The experiment is carried out by adopting a Ti:sapphire laser, and H_2_ and D_2_ molecules (see the methods). These isotopes have attracted extensive interest as a prototype. Figure 2a–c displays the spatially resolved harmonic spectra generated from atomic gas Ar and the hydrogen isotopes H_2_ and D_2_, respectively. Their ionization potentials are very close. The spatially integrated HHG signals are presented by the dash-dotted (Ar), solid (H_2_), and dashed (D_2_) lines in Fig. 2d. One can see that the harmonic intensities from D_2_ are higher than those from H_2_, which is in consistent with previous studies^27–29,36,37^. More importantly, the measured harmonics from H_2_ and D_2_ present obvious frequency shift with respect to those from Ar. As shown in Fig. 2e–h, each harmonic from both H_2_ and D_2_ shows a red shift relative to that from Ar. While for D_2_, the frequency shift is larger than that of H_2_.

### Gas pressure dependence of FM

**Fig. 3:**
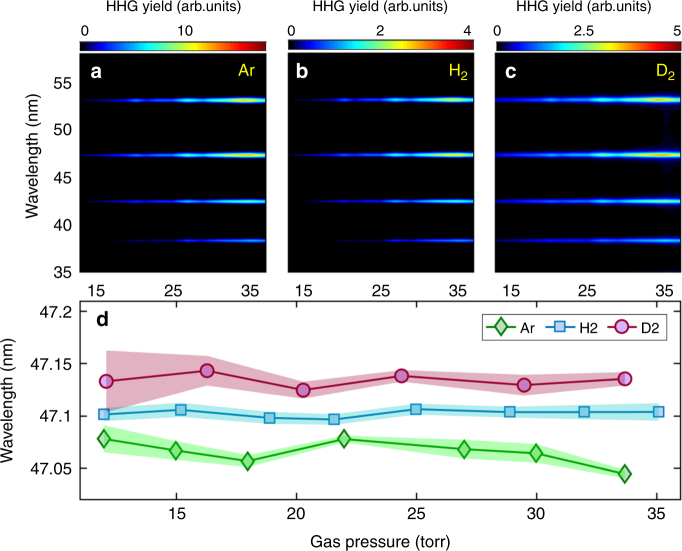
Gas pressure-dependent HHG. **a**–**c** Measured harmonic spectra from Ar (**a**), H_2_ (**b**), and D_2_ (**c**) at different gas pressures. **d** The central wavelength of H17 from Ar (diamonds), H_2_ (squares), and D_2_ (circles) as a function of the gas pressure. Shaded areas in **d** represent the standard deviation of nine independent measurements

HHG in gas medium includes the individual response, as well as the copropagation of laser and harmonic fields. The propagation effect can possibly induce a frequency shift in HHG^38–40^, which depends sensitively on the gas pressure. However, in our experiment the ionizations of the three gases are weak (below 4%), and also the gas pressure is low. Then the frequency shift induced by the propagation effect will be inappreciable. To check this effect, we measured the harmonic spectra generated from Ar, H_2_, and D_2_ at different gas pressures. With the gas pressure changing from 15 to 35 torr, the intensity of each harmonic from these three gases exhibits a quadratic increase, which indicates a good phase matching in our experiment. More than that, the central wavelengths of each harmonic from the three gases are nearly unchanged as shown in Fig. 3a–c. For a clear insight, in Fig. 3d, we present the central wavelength of H17 for Ar (diamonds), H_2_ (squares), and D_2_ (circles) as a function of the gas pressure. The frequency shift of H_2_ and D_2_ relative to that of Ar keeps almost constant as the gas pressure varies. These results indicate that the influence of propagation effect on the harmonic frequency shift is negligible in our experiment. Besides, the experimental conditions used for HHG from H_2_ and D_2_ are exactly the same, the differences in the harmonic spectra can be mainly attributed to the individual response of isotope molecules in the driving laser field.

### Theoretical simulation of FM

It has been reported that the nonadiabatic effect of the time-dependent laser intensity can induce a blue or red shift when HHG is dominant at the leading or trailing edge of the laser pulse^41–43^. For H_2_ (or D_2_), the ionization rate depends sensitively on the internuclear distance *R*^18,19^. Due to the laser-driven nuclear motion, the average internuclear distance at the trailing edge can be larger than that at the leading edge of the laser pulse, which makes the ionization, as well as the HHG signals stronger at the trailing edge, and therefore induces a red shift in the harmonic spectrum. In contrast, since the laser intensity used in our experiment is far smaller than the ionization saturation threshold of Ar, the ionization and HHG of Ar atom mainly occurs at central part of the laser pulse and is symmetric with respect to the pulse center (*t* = 0). Then no obvious net shift exists in the harmonics from Ar. Since the nuclear dynamics is avoided for Ar, it can serve as a benchmark to evaluate the frequency shift of harmonics from the two isotopic molecules. For a given harmonic order, the frequency shift caused by the nonadiabatic effect can be obtained via the time derivative of the laser pulse, namely, $\Delta \omega = \alpha _q\frac{{\partial I(t)}}{{\partial t}}|_{t = t_{\mathrm{i}}}$^44–46^. Here, $I(t) = I_0{\mathrm{exp}}\left( {\frac{{ - 4{\mathrm{ln}}(2)t^2}}{{\tau ^2}}} \right)$ with *I*_0_ = 1.5 × 10^14^ W cm^−2^, *τ* = 30 fs is the envelope of the laser pulse, *t*_i_ is the ionization moment of the given harmonic, and *α*_*q*_ is its phase coefficient, which can be evaluated according to the strong-field approximation (SFA) model^47^. The time derivative with a positive (negative) sign means a blue (red) shift of this harmonic. Owing to the slower nuclear motion of heavier nuclei, the dominant harmonic emission of D_2_ occurs later than that of H_2_ (see Supplementary Note 1 and Supplementary Figure 1). As a result, the HHG from D_2_ experiences a more rapid change of the effective laser intensity (namely, a larger value of $\left| {\frac{{\partial I(t)}}{{\partial t}}} \right|$), which therefore gives rise to a larger red shift in the harmonic spectrum as observed in our experiment. Besides the nonadiabatic effect, the nuclear motion can lead to the variation of the ionization potential and the complex recombination dipole, which may affect the harmonic phase accumulated during the electron excursion and influence the MHOHG^48,49^. To evaluate these influences, we have performed simulations with the modified SFA model^48^, which indicates that the frequency shift induced by these two effects is far smaller than our experimental observations (see Supplementary Note 2 and Supplementary Figures 2 and 3). Moreover, the laser-driven nonadiabatic alignment may also lead to a red shift in MHOHG. We have evaluated this influence by considering the time-dependence of the laser-driven alignment under our experiment condition (see Supplementary Note 3 and Supplementary Figure 4). Our calculations show that the red shift induced by the molecular alignment is about one order of magnitude smaller than our experimental observations. Note also that the fluctuation of the laser carrier-envelope phase, which is not fixed in our experiment, will not affect the measured frequency shift of MHOHG because a multi-cycle laser pulse is used in our experiment. Therefore, the main contribution to the frequency shift shown in Fig. 2 is attributed to the nonadiabatic effect induced by the nuclear motion^32^.

**Fig. 4:**
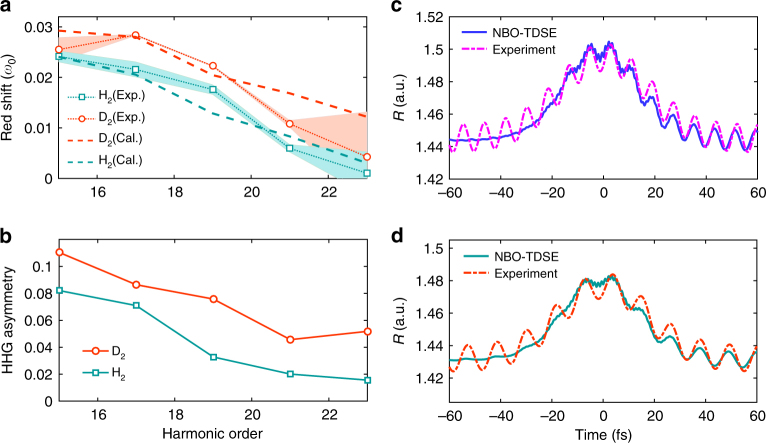
Measured and calculated frequency shifts and nuclear motions of H_2_ and D_2_. **a** Measured and calculated red shift Δ*ω* in MHOHG with respect to harmonics of Ar as a function of the harmonic order. **b** The HHG asymmetry coefficients *η* calculated for H_2_ and D_2_. *η*(*ω*) > 0(<0) means the harmonic emission is more pronounced at the trailing (leading) edge of the laser pulse. Squares and circles are for H_2_ and D_2_, respectively. **c** Calculated (solid line) and experimentally retrieved (dash-dotted line) nuclear vibration of H_2_. **d** Same as **c**, but for D_2_. Shaded areas in **a** represent the standard deviation of nine independent measurements

In Fig. 4a, we present the relative frequency shift of H15-H23 for H_2_ (squares) and D_2_ (circles). The relative frequency shifts gradually decrease as the harmonic order increases. The experiment is also simulated by solving the NBO–TDSE (see the methods). The calculated frequency shifts of H_2_ and D_2_ are presented by the dashed lines in Fig. 4a, which are in agreement with the experimental observations. Some difference in quantity may arise from the uncertainties of experimental parameters. In Fig. 4b, we have calculated the asymmetry coefficients of HHG signals for H_2_ (squares) and D_2_ (circles). Here, the HHG asymmetry is defined as *η*(*ω*) = (*P*_+_(*ω*) − *P*_−_(*ω*))/(*P*_+_(*ω*) + *P*_−_(*ω*))^31^, where $P_ + (w) = {\int}_0^{ + \infty } g(\omega ,t)\mathrm{d}t$ and $P_ - (w) = {\int}_{ - \infty }^0 g(\omega ,t)\mathrm{d}t$ are the amount of harmonic *ω* generated at the trailing and leading edges of the laser pulse, respectively. *g*(*ω*, *t*) is the time-frequency spectrogram calculated with the Gabor transform. In our calculation, the width of the time window used in the Gabor transform is 0.1 fs, which corresponds to a filter with the width of 10*ω*_0_ (*ω*_0_ is laser frequency) in the frequency domain. One can see that for each harmonic order, the harmonic emission is more pronounced at the trailing edge of the laser pulse (namely, *η*(*ω*) > 0). Therefore, all the harmonics exhibit a red shift in the spectrum as shown in Fig. 4a. Moreover, the trend of the red shift agrees qualitatively with the asymmetry coefficients. This agreement suggests that the observed red shift indeed results from the delayed emission of HHG with respect to the center of the laser pulse.

### Monitoring the nuclear dynamics by FM

As mentioned above, the frequency shift mainly arises from the asymmetry of the ionization (and so HHG) with respect to the center of laser pulse (*t* = 0 fs)^32^. Previous studies have shown that the ionization rate of H_2_ (D_2_) is approximately linearly dependent on internuclear distance *R* before it reaches 2 a.u.^50^. Thus the relative frequency shift of the *q*-th harmonic with respect to *qω*_0_ can be estimated as:

1 $$\frac{{\Delta \omega }}{{\omega _0}} = \frac{{\mathop {\sum}\limits_{t_{\mathrm{i}} < 0} R(t_{\mathrm{i}}) - \mathop {\sum}\limits_{t_{\mathrm{i}} > 0} R(t_{\mathrm{i}})}}{{\mathop {\sum}\limits_{t_{\mathrm{i}}} R(t_{\mathrm{i}})}},$$

where *ω*_0_ is the frequency of the driving laser, *t*_i_ is the ionization moment of the electron (contributing to the *q*-th harmonic generation) in each half optical cycle. *t*_i_ < 0 and *t*_i_ > 0 mean the ionization occurs at the leading and trailing edges of laser pulse, respectively. For a given harmonic, *t*_i_ can be calculated according to the three-step model^47,51^. To retrieve the nuclear motion, we consider to employ the commonly used linear harmonic oscillator model^52^ to describe the two-body vibrations of H_2_ and D_2_. The simulations with the NBO–TDSE show that the harmonic oscillator model works well in a low-ionization case. In our experiment, the ionization is below 4%, therefore the harmonic oscillator model is applicable. In the harmonic oscillator model, the potential *V*(*r*) of H_2_ (or D_2_) can be approximatively expressed as $V(r) = V_0 + \frac{k}{2}(r - R_e)^2$, where *V*_0_ and *k* are constants and *R*_e_ is the equilibrium internuclear distance of H_2_ and D_2_. Then the laser-driven nuclear motion can be derived in the form of (for details, see Supplementary Note 4)

2 $$R(t) = A{\mathrm{sin}}({\mathrm{\Omega }}t + \phi ) + BI(t) + R_{\mathrm{e}}.$$

On the right side of Eq. (2), the first term denotes the inherent harmonic vibration, *A*, Ω, and *ϕ* are the corresponding amplitude, frequency, and phase of the vibration. The second term represents the laser-nucleus interaction. Inserting Eq. (2) into Eq. (1), the frequency shift of a specific harmonic can be expressed as a function of *A*,* B*, Ω, and *ϕ*. By fitting the observed frequency shifts of H15-H23 to Eq. (1) with the least square method, the four parameters can be determined. Then the nuclear motion *R*(*t*) can be retrieved. Figure 4c–d shows the retrieved nuclear vibrations (dash-dotted line) of H_2_ and D_2_, respectively. As shown in this figure, the maximum of the retrieved *R*(*t*) of H_2_ is about 1.505 a.u., which is slightly larger than that of D_2_ (1.485 a.u.). Moreover, the retrieved *R*(*t*) of H_2_ oscillates with a period of 8.2 fs. In contrast, it is 11.4 fs for D_2_. The retrieved oscillation periods of H_2_ and D_2_ are very close to the vibrational periods of H_2_ and D_2_ in their ground electronic state (7.5 and 10.6 fs). The ratio of these two retrieved periods is also very close to the expected mass ratio of $\sqrt 2$. The results calculated from the NBO–TDSE are also presented as the solid lines. From Fig. 4c, d, one can see that due to the inherent harmonic vibration of the harmonic oscillator model, the retrieved nuclear motion shows much deeper modulation at the beginning when compared to the simulated one. While with the increase of the laser intensity, the simulated nuclear motion also turns to oscillate after *t* = − 20 fs due to vibrational excitation. Despite the initial oscillation, the main structures of the retrieved nuclear motion *R*(*t*), such as the dynamic range and the overall trend, can agree well with the theoretical predictions in the range of [−20, 20] fs where most of the HHG signals are generated. It should be explained that to compare with the NBO–TDSE calculations, the initial internuclear distance used in the experimental fitting is obtained from the NBO–TDSE simulation [namely, the initial values of the solid lines in Fig. 4c, d]. It is given by the expectation value of *R* with the ground state wavefunction Ψ_0_, namely, 〈Ψ_0_|*R*|Ψ_0_〉. Note that the expectation values 〈Ψ_0_|*R*|Ψ_0_〉 are different for H_2_ and D_2_ due to their different field-free Hamiltonians (depending on the nuclear mass). Moreover, the so-called equilibrium internuclear distance *R*_e_ is defined as the minimum of the BO potential *V*_BO_(*R*), namely, where d*V*_BO_(*R*)/d*R* = 0^53^. Since the BO potential *V*_BO_(*R*) is slightly asymmetric with respect to *R*_e_, the expectation value of *R* obtained from the NBO–TDSE simulation (1.44 a.u. for H_2_ and 1.43 a.u. for D_2_) is slightly different from the so-called equilibrium internuclear distance *R*_e_ (1.4  a.u. for both H_2_ and D_2_^54^). To study the stability of the retrievals, we have also performed the fitting with different harmonic orders or using the known values of the oscillation frequency Ω. The obtained results are all in good agreement with the NBO–TSDE simulations (see Supplementary Note 4 and Supplementary Figures 5 and 6). Considering the simplicity of the harmonic oscillator model and the uncertainty of experimental parameters, the agreement of the retrieved nuclear motions with the TDSE predictions is very satisfying.

## Discussion

In summary, we experimentally observed the red shift in HHG from isotopic molecules H_2_ and D_2_. The red shift is primarily attributed to the laser-driven nuclear motion in H_2_ and D_2_, which strengthens the ionization rate and harmonic emission due to larger internuclear distance *R* and lower *I*_p_ at the trailing edge of the laser pulse. By using a linear harmonic oscillator model, the nuclear vibrations of H_2_ and D_2_ are successfully retrieved from the observed frequency shift. The FM effect in MHOHG is universal, which can be directly applied to other light molecules if the ionization rate is sensitive to nuclear motion. Moreover, in our experiment the molecules are not pre-aligned, the alignment effect is negligible. In principle, the FM technology can be extended to aligned molecules with any alignment angles with respect to the laser polarization. The alignment-angle-dependent FM can not only be used to extract the ultrafast electron-nuclear dynamics, but also be possible to image molecular structure.

In previous studies of AM, the intensity ratios of HHG from isotopic molecules reveal the nuclear dynamics of ${\mathrm{H}}_2^ +$ and ${\mathrm{D}}_2^ +$ within the time window from ionization to recombination in one laser cycle, namely, intracycle dynamics. In contrast, in the present work, the observed frequency shift provides a monitoring of the nuclear vibrations of H_2_ and D_2_ at each ionization moment in the laser pulse, namely, intercycle dynamics. Therefore, FM in MHOHG reveals a different physical process and is complementary with the method of AM^27–29^ for probing the nuclear dynamics. These findings may provide a deep insight into some of the most fundamental events in chemistry and facilitate the development of HHS.

## Methods

### Experimental methods

The experiment is performed by using a commercial Ti:sapphire laser system (Legend Elite-Duo, Coherent, Inc.), which delivers the 30 fs, 800 nm pulses at a repetition rate of 1 kHz. The output laser pulse is focused to a 2-mm-long gas cell by a 600-mm focal-length lens. In Fig. 2, the stagnation pressure of the gases is 30 torr and the gas cell is placed 2 mm after the laser focus to ensure the phase matching of the short quantum path. The laser energy used in our experiment is maintained at 1.5 mJ and the corresponding intensity is estimated to be 1.5 × 10^14^ W cm^−2^. The generated harmonic spectrum is detected by a homemade flat-field soft x-ray spectrometer consisting of a flat-field grating (1200 grooves mm^−1^) and a slit with a width of about 0.1 mm and height of 15 mm. High-order harmonics are dispersed by the grating and imaged onto the microchannel plate (MCP) fitted with a phosphor screen. The image on the screen is read out by a CCD camera.

To accurately evaluate the frequency shift in MHOHG, we have calibrated the spectrometer by using the atomic lines of carbon in terms of a procedure similar to that in refs. ^55,56^. The atomic lines are produced by focusing several millijoules of the driving laser pulse to interact with a 0.5-mm-thick graphite sheet placed at the position where HHG occurs. We record the generated atomic lines and read their coordinates on the phosphor screen. By assigning the observed atomic lines to the known literature data of carbon, we can then achieve the calibration of the spectrometer. Details of the calibration are provided in Supplementary Methods.

### Theoretical methods

To simulate the HHG process and nuclear dynamics of H_2_ and D_2_, we numerically solve the NBO–TDSE with one active electron^27,57^. Since the electron and nuclear motions follow the linearly polarized laser field, we adopt the one-dimensional model,

$$i\frac{{\partial \Psi (z,R,t)}}{{\partial t}} = [H_{\mathrm{e}}(t) + H_{\mathrm{n}}(t) - E(t)z]\Psi (z,R,t),$$

$$H_{\mathrm{n}} = - \frac{1}{{2\mu }}\frac{{\partial ^2}}{{\partial R^2}} + \frac{1}{R},$$

$$H_{\mathrm{e}} = - \frac{1}{2}\frac{{\partial ^2}}{{\partial z^2}} + V_{{\mathrm{en}}}(R,z),$$

3 $$V_{{\mathrm{en}}}(R,z) = - \frac{{Z(R,|z + R/2|)}}{{\sqrt {(z + R/2)^2 + 0.5} }} - \frac{{Z(R,|z - R/2|)}}{{\sqrt {(z - R/2)^2 + 0.5} }} + V_{{\mathrm{BO}}}^ + (R).$$

Here, *z* is the electron coordinate, *R* is the internuclear distance, and *E*(*t*) is the driving laser field. *H*_n_ and *H*_e_ are the Hamiltonians for the nuclei and electron, respectively. *V*_en_(*R*, *z*) is the Coulomb potential of the electron–nucleus interaction. *μ* is the reduced mass of two nuclei and $V_{{\mathrm{BO}}}^ + (R)$ is the lowest BO potential of H$_2^ +$. In order to faithfully mimic the nuclear dynamics of H_2_ and D_2_, we have adopted an effective nuclear charge $Z(R,\xi ) = \left[ {1 + e^{ - \xi ^2/\sigma ^2(R)}} \right]/2$, where *σ*(*R*) is an *R*-dependent screening parameter. By adjusting the parameter *σ*(*R*) at each internuclear distance, the energy of the ground electronic state of hydrogen and hydrogen ion can be well reproduced.

The TDSE is solved using the *B*-spline method with Crank–Nicolson scheme^58^. The initial wave function is taken as the ground state of the hydrogen molecule calculated using the imaginary-time propagation method. The wave function is numerically discretized in the grid with a size of −50 ≤ *z* ≤ 50 a.u. and *R* ≤ 18 a.u. The discrete steps for the electron coordinate and nuclear coordinate are *δz* = 0.1 and *δR* = 0.05 a.u., respectively. The temporal step is *δt* = 0.05 a.u. Then the harmonic spectrum can be obtained by performing the Fourier transform of the laser-induced electron dipole moment *d*(*t*) = 〈Ψ(*z*,*R*,*t*)|*z*|Ψ(*z*,*R*,*t*)〉. The expectation of the internuclear distance is calculated by $R(t) = \frac{{\left\langle {\Psi \left( {z,R,t} \right)\left| R \right|\Psi \left( {z,R,t} \right)} \right\rangle }}{{\left\langle {\Psi \left( {z,R,t} \right)\Psi \left( {z,R,t} \right)} \right\rangle }}$^59^.

To confirm the results of the one-electron model, we have also solved the NBO–TDSE with two electrons^22,23^, which reads

$$i\frac{{\partial \psi (z_1,z_2,R,t)}}{{\partial t}} = \left[ {H_{\mathrm{e}} + H_{\mathrm{n}} + E(t)(z_1 + z_2)} \right]\psi (z_1,z_2,R,t),$$

$$H_{\mathrm{n}} = - \frac{1}{{2\mu }}\frac{{\partial ^2}}{{\partial R^2}} + \frac{1}{R},$$

$$H_{\mathrm{e}} = - \frac{1}{2}\frac{{\partial ^2}}{{\partial z_1^2}} - V_{{\mathrm{en}}}(z_1) - \frac{1}{2}\frac{{\partial ^2}}{{\partial z_2^2}} - V_{{\mathrm{en}}}(z_2) + V_{{\mathrm{ee}}}(z_1 - z_2),$$

$$V_{{\mathrm{en}}}(z_i) = \frac{1}{{\sqrt {(z_i + R/2)^2 + \beta (R)^2} }} + \frac{1}{{\sqrt {(z_i - R/2)^2 + \beta (R)^2} }},$$

4 $$V_{{\mathrm{ee}}} = \frac{1}{{\sqrt {(z_1 - z_2)^2 + \gamma (R)^2} }}.$$

*H*_n_ and *H*_e_ are the Hamiltonians for the nuclei and electrons. *V*_en_(*z*_*i*_) and *V*_ee_ are the Coulomb potential for the electron–nucleus and electron–electron interactions. *β*(*R*), *γ*(*R*) are the *R*-dependent softening parameters. Due the huge computation of the two-electron model, we calculate only the harmonic spectrum for H_2_. The obtained red shift is consistent with the one-electron model, as well as the experimental observations.

### Data availability

All the data that support the findings of this study are available from the corresponding author upon reasonable request.

## Electronic supplementary material

Supplementary Information(PDF 1032 kb)
