# Supplementary material for: Monitoring ultrafast vibrational dynamics of isotopic molecules with frequency modulation of high-order harmonics
Source: Nat Commun. 2018 Mar 16;9:1108. doi: 10.1038/s41467-018-03568-3 (PMC5856770; doi:10.1038/s41467-018-03568-3)
Supplement: Supplementary file 1 — Supplementary Information(PDF 1032 kb) [file 41467_2018_3568_MOESM1_ESM.pdf]

Supplementary Information

Monitoring Ultrafast Vibrational Dynamics of Isotopic Molecules with Frequency Modulation of High-order Harmonics

He *et al.*

Supplementary Figures

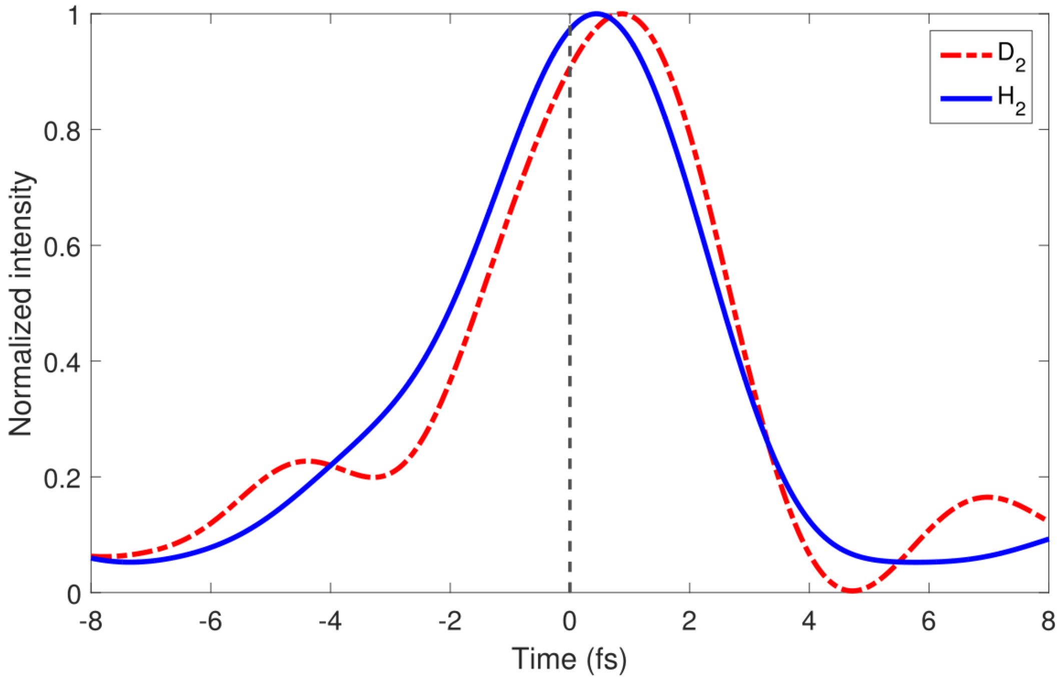

Supplementary Figure 1: Temporal profiles of HHG from H_2 and D_2. Temporal profiles of H17 simulated for D_2 (dashed line) and H_2 (solid line).

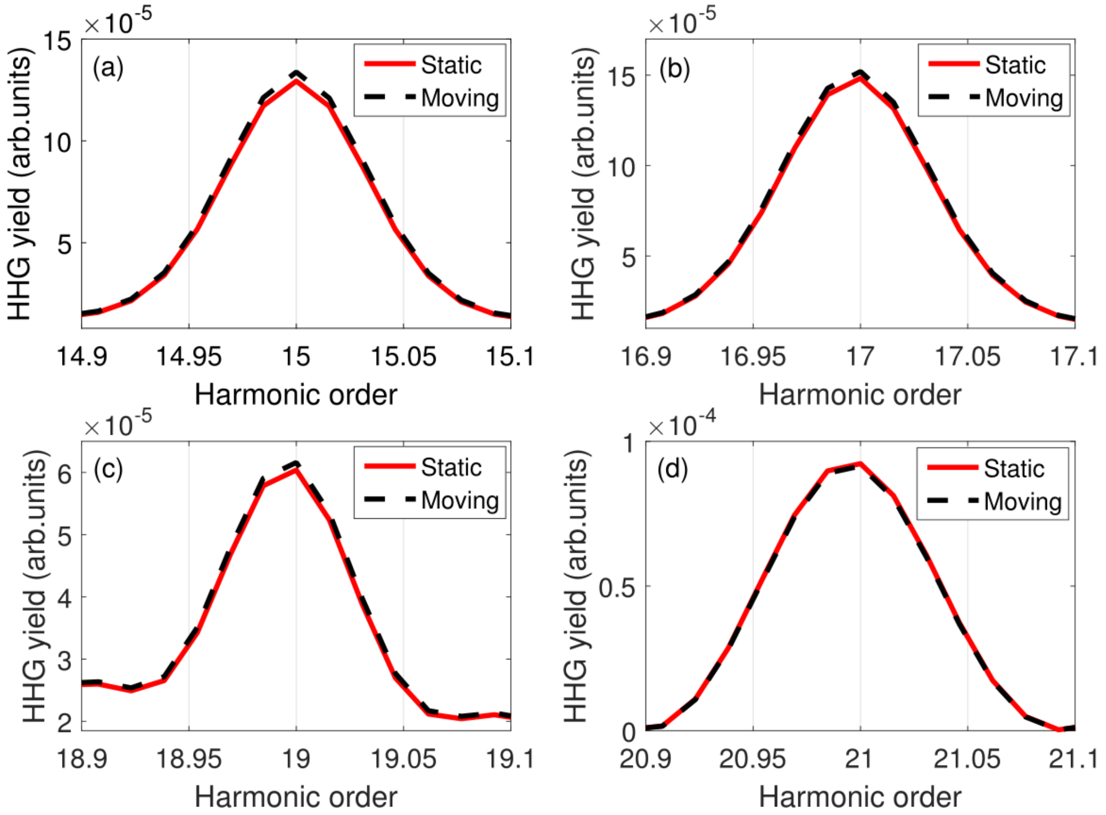

Supplementary Figure 2: Effect of the variation of the recombination matrix element on HHG from H_2. Harmonic signals of H15-H21 from H_2 calculated by using the modified SFA model. Solid line is for the nuclear distance fixed at *R*=1.44 a.u. Dashed line is obtained for the recombination matrix element changing with the nuclear motion *R*(t). (a)-(d) are the results of H15, H17, H19, and H21, respectively.

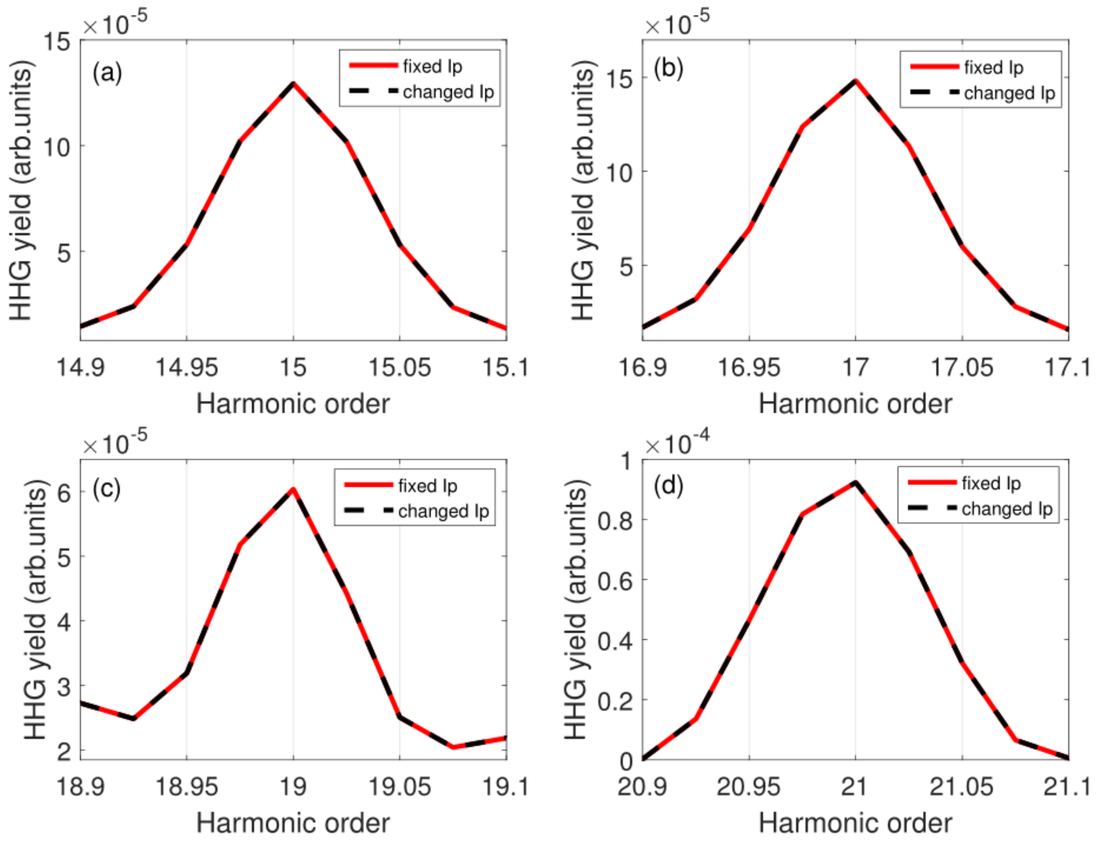

Supplementary Figure 3: Effect of the variation of the ionization potential on HHG from H_2. Harmonic signals of H15-H21 from H_2 calculated by using the SFA model with the ionization potential fixed at 15.43 eV (solid line) and changed with $R(t)$ (dashed line). (a)-(d) are the results of H15, H17, H19, and H21, respectively.

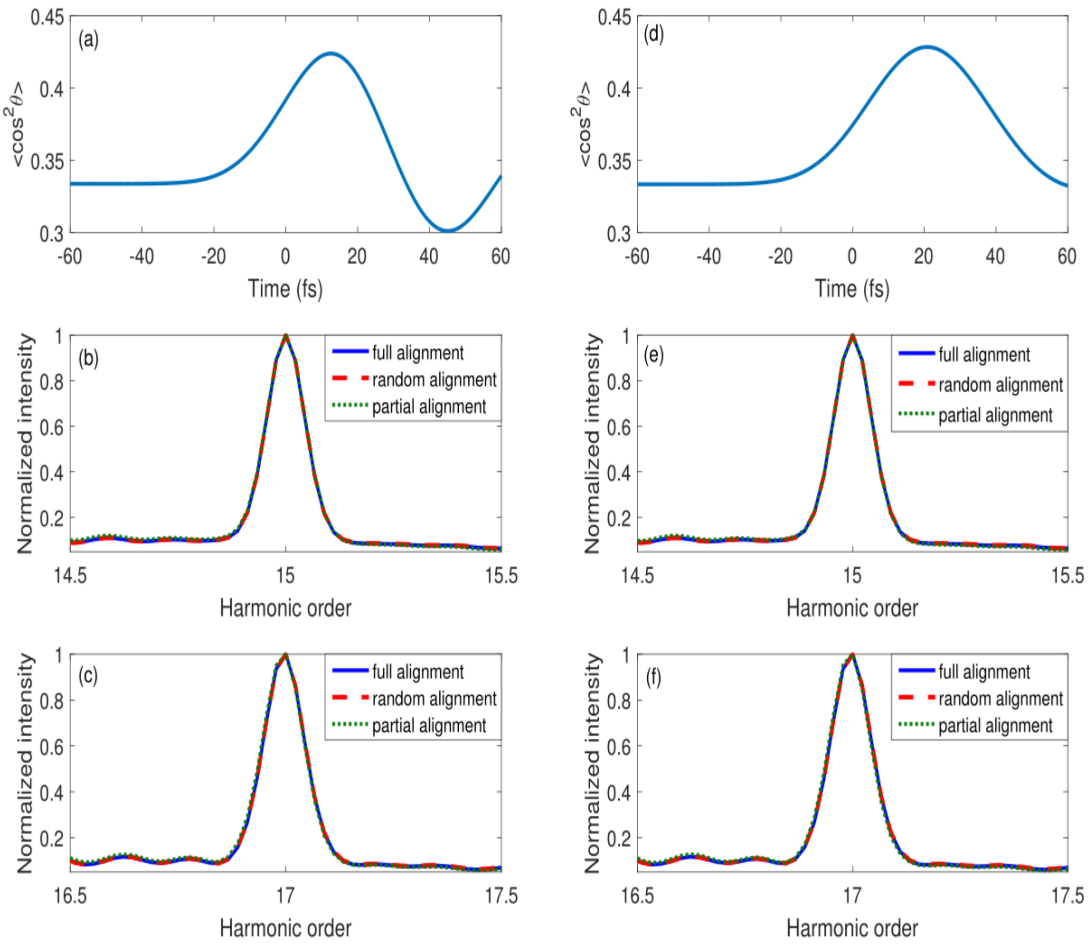

Supplementary Figure 4: Alignment effect on HHG of H_2 and D_2. (a) Non-adiabatic alignment degree $<\cos^2\theta>$ of H_2 in the driving laser pulse. (b)-(c) Harmonic signals of H15 and H17 calculated with the molecules fully (along laser polarization direction), randomly, and partially (under the alignment degree in (a)) aligned. (d)-(f) Same as (a)-(c), but for D_2.

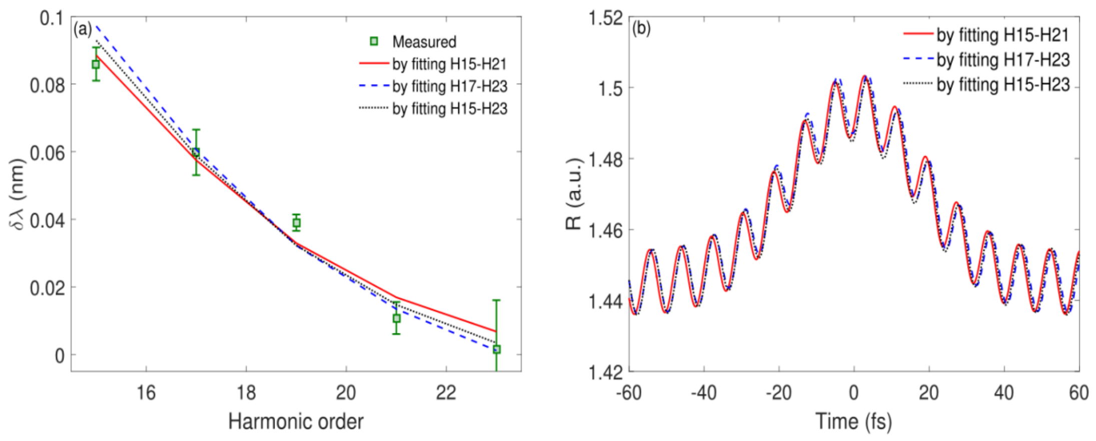

Supplementary Figure 5: Experimental retrievals with different harmonic orders. (a) Frequency shift and (b) nuclear motion of H_2 reproduced by using the measured frequency shifts of H15-H21 (solid line), H17-H23 (dashed line), and H15-H23 (dotted line). Error bars in (a) represent the standard deviation of the experimental measurement.

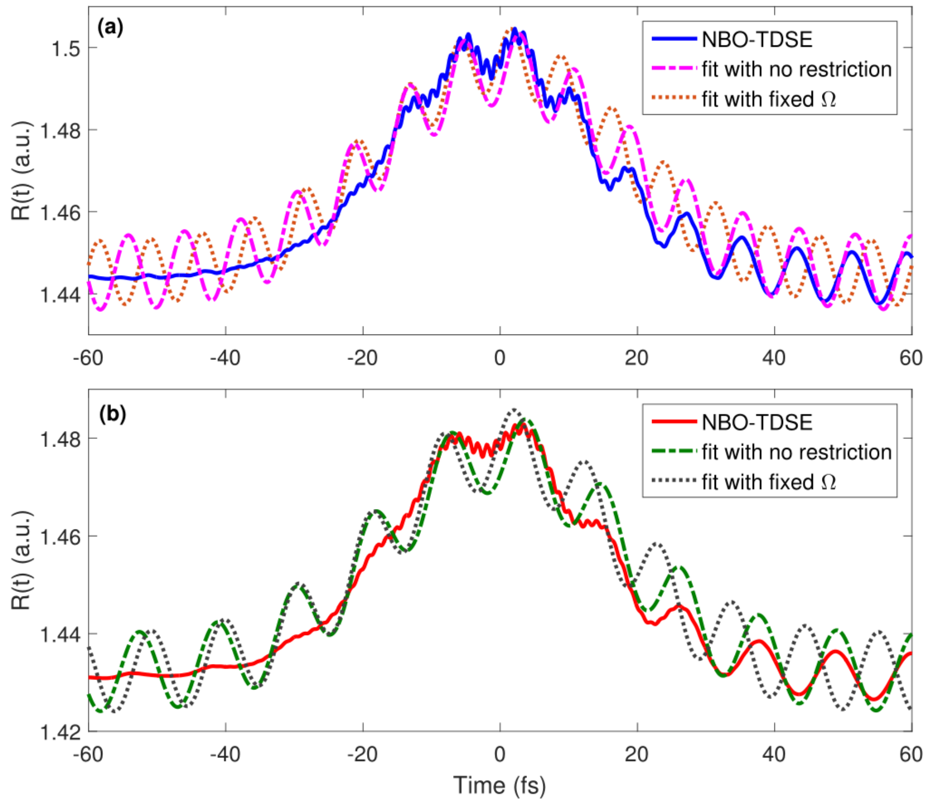

Supplementary Figure 6: Experimental retrievals with reduced parameters. Calculated (solid lines) and experimentally retrieved (dash-dotted and dotted lines) nuclear vibration of (a) H_2 and (b) D_2. Dash-dotted lines are the results obtained by fitting the experimental data (H15-H23) with no restriction, i.e., the retrievals in the main text. Dotted lines are the results fitted with fixed Ω.

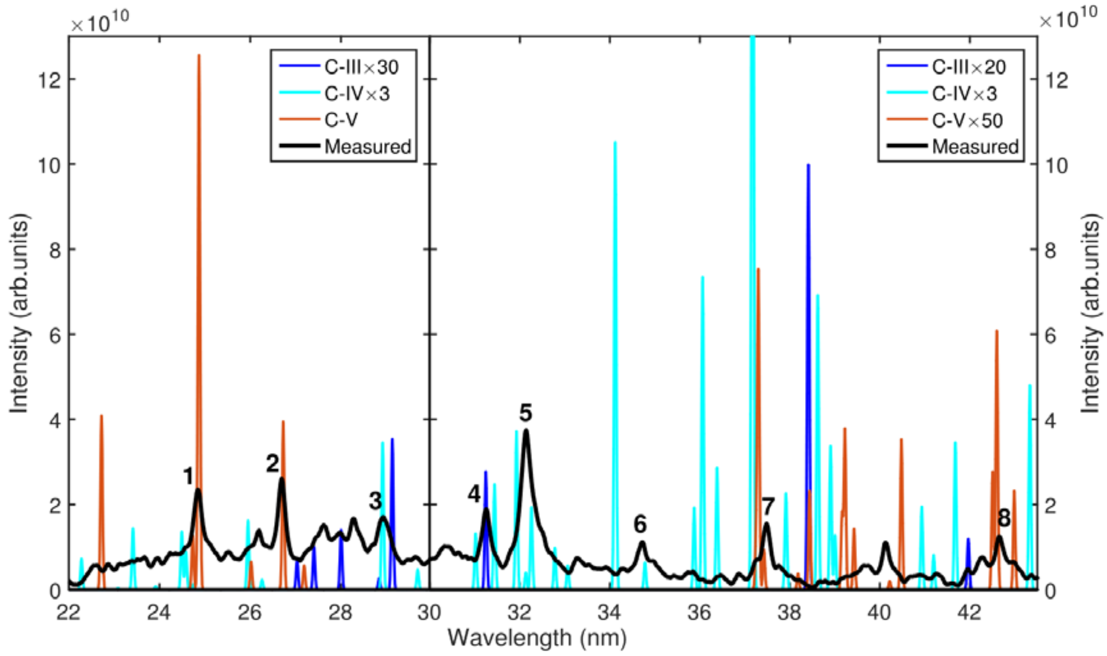

Supplementary Figure 7: Calibration of high harmonic spectrometer. Measured atomic spectrum (black) of Carbon and the atomic lines of Carbon-III through V tabulated by NIST. In the left and right panels, the NIST data are multiplied by different factors (as labeled) for better comparison with the measured spectrum.

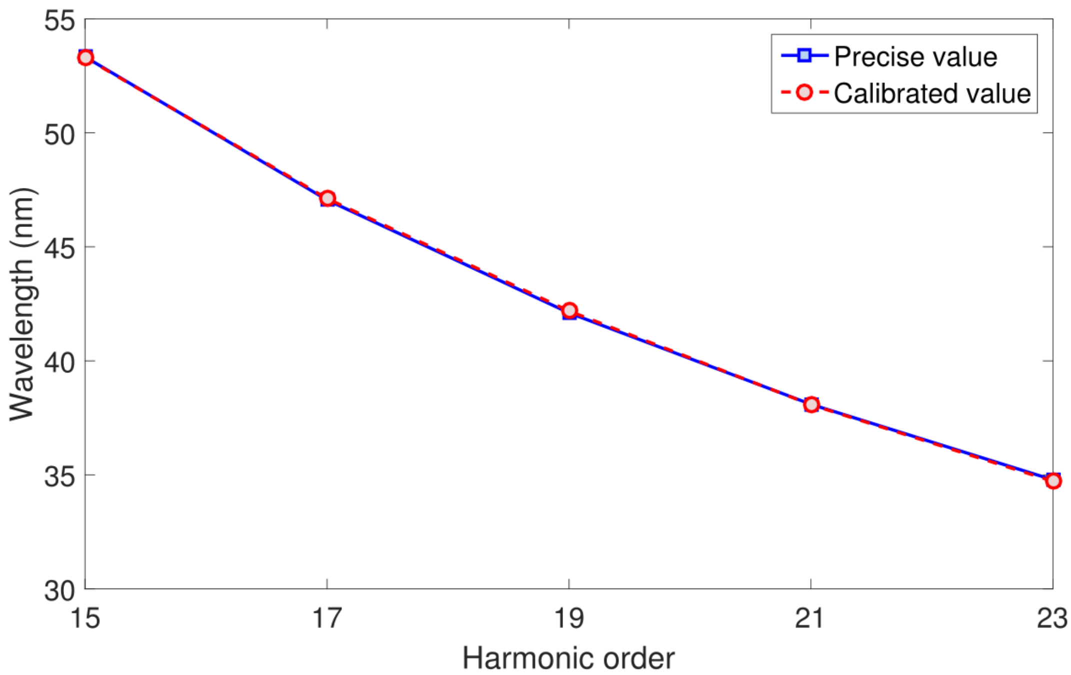

Supplementary Figure 8: Calibrated wavelengths of HHG of Ar. Calibrated wavelengths (circles) of harmonics (H15-H23) from Ar. For comparison, the precise values of the harmonic wavelengths are also presented (squares).

Supplementary Notes

Supplementary Note 1: Temporal profiles of HHG from H_2 and D_2

In our work, the observed red shift in MHOHG is ascribed to the dominant HHG from the stretched H_2 and D_2 at the trailing edge of the laser pulse. To demonstrated this point, we have simulated the temporal profiles of HHG from H_2 and D_2, respectively. Here, the HHG temporal profile is obtained by superposing HHG signals in a narrow band ($\sim 1\omega_0$) centered at the given harmonic order [1]. In Supplementary Figure 1, we show the result of H17 as an example. One can see that HHG from both H_2 and D_2 are dominated at the trailing edge of the laser pulse (t>0). Therefore, due to laser-induced nonadiabatic effect, HHG from these two isotopes are both red-shifted. Moreover, the dominant HHG from D_2 is later than that from H_2, thus leading to a larger red shift in D_2. This result is consistent with our explanation in the main text.

Supplementary Note 2: Effect of the variations of the recombination matrix element and ionization potential on HHG from H_2 and D_2

Besides the nuclear motion induced nonadiabatic effect, the variation of the recombination matrix element induced by nuclear motion can also influence the MHOHG. To study this effect, we have calculated the molecular harmonic generation with the modified SFA model [2]. The recombination matrix element is given by $\mathbf{d}_{\rm rec}(\mathbf{k})=\langle\Psi_0(\mathbf{r})|\mathbf{r}|\Psi_c(\mathbf{r})\rangle$, where $\Psi_0(\mathbf{r})$ is the ground-state wave function and $\Psi_c(\mathbf{r})$ is the continuum wave function. In our calculation, we adopt the two-center Coulomb wave, which is the solution of the two-body Coulomb continuum problem by including the main Coulomb effects on the recolliding wave packet [3], to describe the continuum states. Since both the $\Psi_0(\mathbf{r})$ and $\Psi_c(\mathbf{r})$ depend on the internuclear distance, the nuclear motion in the laser filed will therefore lead to the variation of the recombination matrix element. In our work, the nuclear motion $R(t)$ can be calculated from the NBO-TDSE. Then the R-dependent recombination matrix element can be obtained by $\mathbf{d}_{\rm rec}(\mathbf{k},t)=\langle\Psi_0(R(t),\mathbf{r})|\mathbf{r}|\Psi_c(R(t),\mathbf{r})\rangle$. In Supplementary Figure 2, we show the harmonic signals of H15-H21 for H_2 calculated by using the R-dependent recombination matrix element (dashed lines). For comparison, the results calculated with the fixed nuclear distance R=1.44 a.u. are also presented (solid lines). One can clearly see that, the R-dependent recombination matrix element makes a difference in the strength of each high harmonic, but does not induce a distinct frequency shift ($\delta\lambda\sim$0.001 nm). As comparing Supplementary Figure 2 with Figure 2 of our manuscript, the frequency shifts for all these harmonics are unapparent and negligible compared with the experimental results. Note that we also performed the simulation by describing the continuum state $\Psi_c(R(t),\mathbf{r})$ with the plane wave as in the original SFA model [4]. The frequency shift is negligible compared with the experimental observations too.

On the other hand, we further consider the effect of nuclear motion during the electron excursion. The nuclear motion will lead to the variation of the ionization potential and can affect the harmonic phase accumulated during the electron excursion, which can be expressed by $S = \int_{t_\mathrm{i}}^{t_\mathrm{r}} (\frac{[p-A(t)]^2}{2} + I_\mathrm{p})dt$[4], where p is the canonical momentum, $A(t)$ is the vector potential of the laser field, and I_p is the ionization potential. However, in our experiment, the dynamic range of the nuclear motion is very small. The change of the ionization potential is inappreciable, which will not affect the harmonic phase significantly. To examine this effect, we have considered the variation of ionization potential during the electron excursion and calculated the HHG from H_2 with the ionization potential I_p changing with $R(t)$. The simulated results are shown in Supplementary Figure 3 (dashed lines). The results calculated with fixed I_p are also presented (solid lines) for comparison. One can see that harmonic signals calculated with R-dependent I_p are almost the same as those with fixed I_p. The influence of the variation of ionization potential during the electron excursion is even smaller than the recombination effect. Therefore, the variations of the recombination matrix element as well as the nuclear motion during the electron excursion are not the main contribution to the frequency shift observed in our experiment.

Supplementary Note 3: Alignment effect on the frequency shift in HHG from H_2 and D_2

Here we discuss the influence of the laser-driven non-adiabatic molecular alignment on the frequency shift in our experiment. The laser-driven nonadiabatic alignment will make more molecules aligned at t>0, which could produce more HHG at the trailing edge and may also lead to a red shift in MHOHG. To evaluated this effect, we treat the molecule as a rigid rotor [5, 6]. When the molecule is placed in a short laser field, the laser will excite a rotational wave packet (coherent superposition of rotational states) in the molecule. The evolution of the rotational wave packet in the laser field can be modeled by the following time-dependent Schrödinger equation [5, 6]

$$i\frac{\partial\Psi_{JM}(\theta,\varphi,t)}{\partial t} = \left[B_\mathrm{e}\hat{J}^2 - \frac{E(t)^2}{2}(\alpha_\parallel\cos^2\theta + \alpha_\perp\sin^2\theta)\right]\Psi_{JM}(\theta,\varphi,t), \qquad (1)$$

where $E(t)$ is the laser field, B_e is the rotational constant, $\hat{J}$ is the rotation operator, $\alpha_\parallel$ and $\alpha_\perp$ are the anisotropic polarizabilities in parallel and perpendicular directions with respect to the molecular axis, respectively. Eq. (1) can be solved with the split-operator method [7] for each initial rotational state $|JM\rangle$. By assuming a Boltzmann distribution of the rotational levels at the initial time, we can obtain the time-dependent alignment distribution as

$$\rho(\theta,t)=\sum_{JM}\Gamma_{JM}\left|\Psi_{JM}(\theta,\varphi,t)\right|^2, \quad (2)$$

where Γ_{JM} is the weight according to the Boltzmann distribution. Once the alignment distribution is obtained, we can then calculate the time-dependent dipole for HHG. At each moment, the total dipole moment D(t) for HHG can be given by the coherent superposition of the alignment-angle-dependent dipole moment $d(\theta,t)$ weighted by the time-dependent alignment distribution $\rho(\theta,t)$, i.e.,

$$D(t)=2\pi\int_0^{\pi} d(\theta,t)\rho(\theta,t)\sin\theta\, d\theta. \quad (3)$$

Here the alignment-angle-dependent dipole $d(\theta,t)$ is calculated by using the SFA model with the nuclear distance fixed at R=1.44 a.u to exclude the effect of nuclear motion. In Supplementary Figure 4 (a) and (d), we show the calculated alignment degrees $\langle\cos^2\theta\rangle$ for H_2 and D_2, respectively. The laser parameters are the same as mentioned in the main text. It's well known that H_2 and D_2 molecules are usually difficult to align due to their ultrashort alignment times (the time for molecule to rotate by 90°). In our work, the HHG experiment is performed at room temperature (~298 K), which makes it more difficult for molecular alignment. Thus the maximum alignment degrees of both H_2 and D_2 are only 0.43, which is very close to the random alignment case (0.33). Furthermore, in Supplementary Figure 4 (b-c) and (e-f), we have calculated harmonic signals for H_2 and D_2 with the molecules fully aligned along the laser polarization direction (solid line), randomly aligned (dashed line), and partially aligned (dotted line) under the time-dependent alignment degrees in (a) and (d). For the case of full (or random) alignment, the alignment distribution $\rho(\theta,t)$ is time-independent. Therefore, the calculated harmonics show no net red shift. While for the partial alignment, the alignment degree is larger at t>0 for both H_2 and D_2, which may lead to a red shift in the generated harmonics. However, we find that the red shift induced by the alignment effect is rather small, which is about one order of magnitude smaller than our experimental observations. Such a weak alignment effect is partly due to the small alignment degrees in our experiment. More importantly, it's because the influence of the molecular alignment depends on the molecular internuclear distance [8]. For H_2/D_2 near the equilibrium internuclear distance (i.e., in our experiment), the ionization rates as well as the HHG change unnoticeably with the alignment angles. Therefore, the alignment effect in our experiment is insignificant and can be ruled out.

Supplementary Note 4: Linear harmonic oscillator model for H_2 and D_2

We employ a linear harmonic oscillator model [9] to describe the nuclear motions of H_2 and D_2. In this model, the potential $V(r)$ of H_2 or D_2 can be approximately expressed as $V(r)=V_0+k(r-R_e)^2/2$ (a typical potential of linear harmonic oscillator),

where V_0 and k are constants and R_e is the equilibrium internuclear distance of H_2/D_2. Then the laser-driven nuclei of H_2 or D_2 can be treated as a forced harmonic oscillator, and the nuclear motion $R(t)$ can be described by

$$m\frac{d^2R}{dt^2} + k(R - R_e) = F(t), \tag{4}$$

where m is the nuclear mass, $F(t)$ is the laser-induced force, which is proportional to the laser intensity $I(t)$. In our experiment, the laser intensity is given by $I(t)=I_0\exp(-4\ln(2)t^2/\tau^2)$ with $I_0= 1.5\times10^{14}$ Wcm^{-2} and τ=30 fs. The formal solution of $R(t)$ then can be assumed in the form of $R(t)=A\sin(\Omega t+\phi)+G(t)+R_e$. On the right side of this equation, the first term denotes the inherent harmonic vibration of the nucleus, A, Ω, and ϕ are the corresponding amplitude, frequency, and phase of the vibration. The second term $G(t)$ represents the laser-nucleus interaction, which can be derived as (for calculation details, see Refs. [10–12]):

$$G(t) = \frac{\sqrt{2}}{\alpha}|Z|\cos[\Omega t - \mathrm{ang}(Z)], \tag{5}$$

where $\Omega = \sqrt{k/m}$, $\alpha = \sqrt{m\Omega}$, and $Z = \frac{i}{\sqrt{2\alpha}}\int F(t)e^{it}dt$. We have numerically demonstrated that with a Gaussian-shaped $I(t)$, the $G(t)$ is approximately proportional to $I(t)$. Then we can reasonably assume

$$R(t) = A\sin(\Omega t + \phi) + BI(t) + R_e. \tag{6}$$

Inserting Eq. (6) to the expression of the relative frequency shift evaluated by the ionization asymmetry (as has been given out in the main text), i.e.,

$$\frac{\Delta\omega}{\omega_0} = \frac{\sum_{t_i<0} R(t_i) - \sum_{t_i>0} R(t_i)}{\sum_{t_i} R(t_i)} \tag{7}$$

we can obtain the frequency shift $\Delta\omega$ as a function of A, B, Ω, ϕ and t_i. For simplicity, in the following we abbreviate $\Delta\omega=H(A, B, \Omega, \phi, t_i)$. Here, $H(A, B, \Omega, \phi, t_i)$ is a known function, t_i is ionization moment of the electrons in each half optical cycle. For a given harmonic, t_i can be calculated according to the three-step model [4]. Since the values of $\Delta\omega$ of H15-H23 from H_2 and D_2 have been observed in experiment, we can then determine the four parameters A, B, Ω, and ϕ by fitting the experiment data to $\Delta\omega=H(A, B, \Omega, \phi, t_i)$ with the least square method. Once A, B, Ω, and ϕ are obtained, we can then retrieve the nuclear motion as $R(t)=A\sin(\Omega t+\phi)+BI(t)+R_e$ for both H_2 and D_2.

With the least square method, the solutions of the four parameters are unique when 4 (or more) specific harmonics are used, e.g., H15-H21. A different choice of the harmonic orders could make a difference in the solution. To study the stability of our

results, we have extracted the four parameters by using the experimental data of H15-H21, H17-H23, and H15-H23, respectively. The retrieved frequency shifts and nuclear motion of H_2 are shown in Supplementary Figure 5. One can see that, in all these three cases, the retrieved nuclear motions are nearly the same, with only minor difference. This indicates that our results are robustly convergent.

Moreover, to avoid the over-parameterized problem, we have also performed the data fitting with the vibration frequency **Ω** fixed to its literature values (it's 0.0203 a.u. for H_2 and 0.0143 a.u. for D_2. The corresponding vibrational periods are 7.5 fs and 10.6 fs, respectively) to reduce the number of the free parameters. The results are shown as the dotted lines in Supplementary Figure 6, which agree essentially with the NBO-TDSE simulations (solid lines) in the range of [−20, 20] fs where most of the HHG signals are generated, as well the results retrieved with no restriction (dash-dotted lines), i.e., the retrievals in the main text. Such an agreement indicates that our fitting with 5 data points to determine the 4 unknown parameters is still reliable.

Supplementary Methods: Calibration of high harmonic spectrometer

To accurately evaluate the frequency shift in HHG from H_2 and D_2, one needs to calibrate the high harmonic spectrometer with high precision. In our experiment, we have calibrated the spectrometer by using the atomic spectrum of Carbon in terms of the procedure introduced in Refs. [13, 14]. In detail, we focus several millijoules of the driving laser pulse to a piece of graphite sheet (0.5 mm thick) that is placed at the position where HHG occurs to produce the atomic spectrum of Carbon. We record the generated atomic lines and read their coordinates on the phosphor screen. By assigning the observed atomic lines (as labeled in Supplementary Figure 7) to the known NIST data of Carbon, we can build a link between the coordinates and wavelengths, and achieve the calibration of the spectrometer. Supplementary Figure 7 shows the calibrated atomic spectrum of Carbon (black) as well as the atomic lines of Carbon III-V obtained from NIST data. Note that many of atomic lines given by NIST are very close together. In Supplementary Figure 7, we have assumed that each atomic line had a gaussian shape with an amplitude given by the relative amplitudes from NIST. Once the spectrometer is calibrated, we can then determine the central wavelengths of harmonics generated from Ar, H_2 and D_2 according to their coordinates on the phosphor screen. In Supplementary Figure 8, we show the calibrated central wavelengths (circles) of H15-H23 for Ar. For comparison, the precise values (800/*n* with *n* the harmonic order) are also presented (squares). One can see that for each harmonic order, the calibrated result is very close to the precise value. This suggests that the harmonics generated from Ar are nearly not shifted, thus can serve as the benchmark to evaluate the frequency shift of HHG from H_2 and D_2.

Supplementary References

[1] P. Antoine, A. L'Huillier, and M. Lewenstein, Attosecond pulse trains using high–order harmonics, *Phys. Rev. Lett.* **77**, 1234-1237 (1996).
[2] Anh-Thu Le, T. Morishita, R. R. Lucchese, and C. D. Lin, Theory of high harmonic generation for probing time-resolved large-amplitude molecular vibrations with ultrashort intense lasers, *Phys. Rev. Lett.* **109**, 203004 (2012).
[3] M. F. Ciappina, C. C. Chirila, and M. Lein, Influence of Coulomb continuum wave functions in the description of high-order harmonic generation with H_2^+, *Phys. Rev. A* **75**, 043405 (2007).
[4] M. Lewenstein, Ph. Balcou, M. Yu. Ivanov, A. L'Huillier, and P. B. Corkum, Theory of high-harmonic generation by low-frequency laser fields, *Phys. Rev. A* **49**, 2117-2132 (1994).
[5] H. Stapelfeldt, and T. Seideman, Colloquium: Aligning molecules with strong laser pulses, *Rev. Mod. Phys.* **75**, 543-557 (2003).
[6] Anh-Thu Le, R. R. Lucchese, S. Tonzani, T. Morishita, and C. D. Lin, Quantitative rescattering theory for high-order harmonic generation from molecules, *Phys. Rev. A* **80**, 013401 (2009).
[7] S. Saugout, E. Charron, and C. Cornaggia, H_2 double ionization with few-cycle laser pulses, *Phys. Rev. A* **77**, 023404 (2008).
[8] A. D. Bandrauk, and J. Ruel, Charge-resonance-enhanced ionization of molecular ions in intense laser pulses: Geometric and orientation effects, *Phys. Rev. A* **59**, 2153-2162 (1999).
[9] L. D. Landau, and E. M. Lifshitz, *Quantum Mechanics* (Pergamon, New York, 1958).
[10] E. P. Wigner, On the quantum correction for thermodynamic equilibrium, *Phys. Rev.* **40**, 749-759 (1932).
[11] E. Kerner, Note on the forced and damped oscillator in quantum mechanics, *Can. J. Phys.* **36**, 371-377 (1958).
[12] P. G. L. Leach, Note on the time-dependent damped and forced harmonic oscillator, *Am. J. Phys.* **46**, 1247-1249 (1978).
[13] A. D. Shiner *et al.*, Probing collective multi-electron dynamics in xenon with high-harmonic spectroscopy, *Nat. Phys.* **7**, 464-467 (2011).
[14] J. P. Farrell, B. K. McFarland, P. H. Bucksbaum, and M. Gühr, Calibration of a high harmonic spectrometer by laser induced plasma emission, *Opt. Express* **17**, 15134-15144 (2009).
